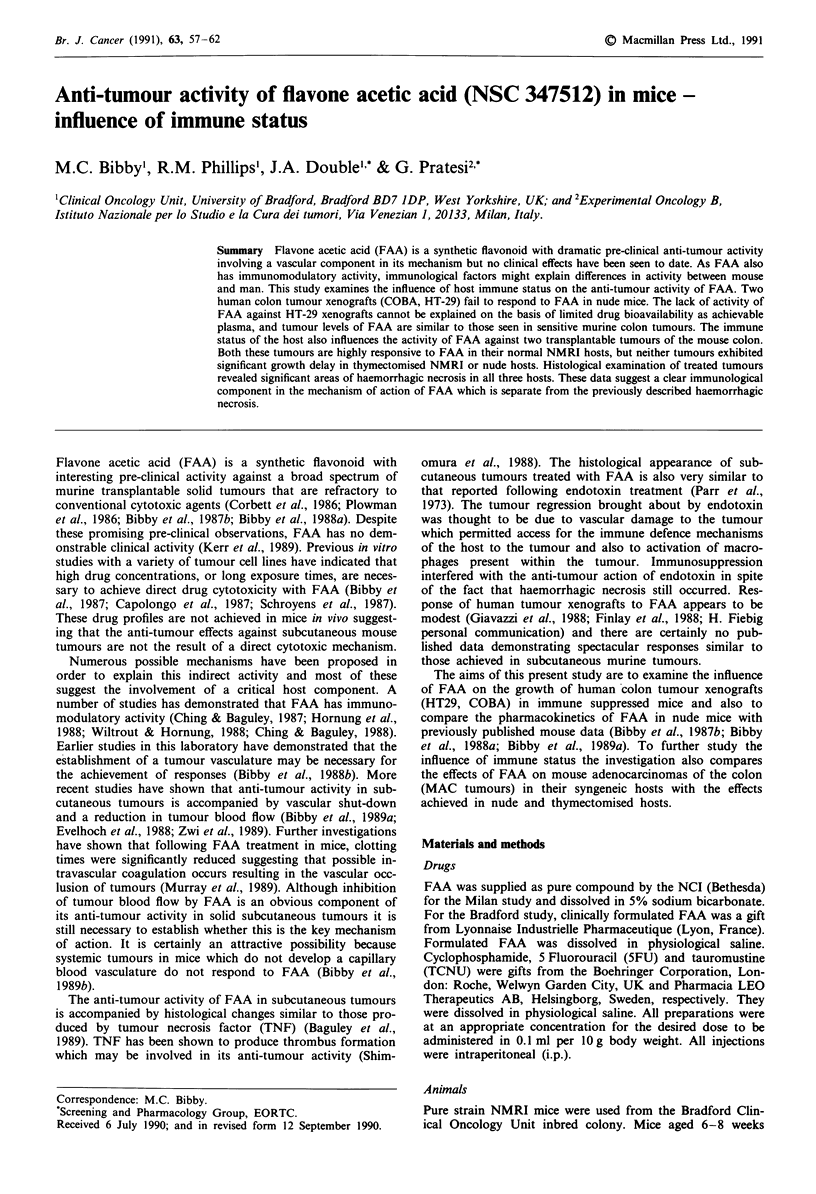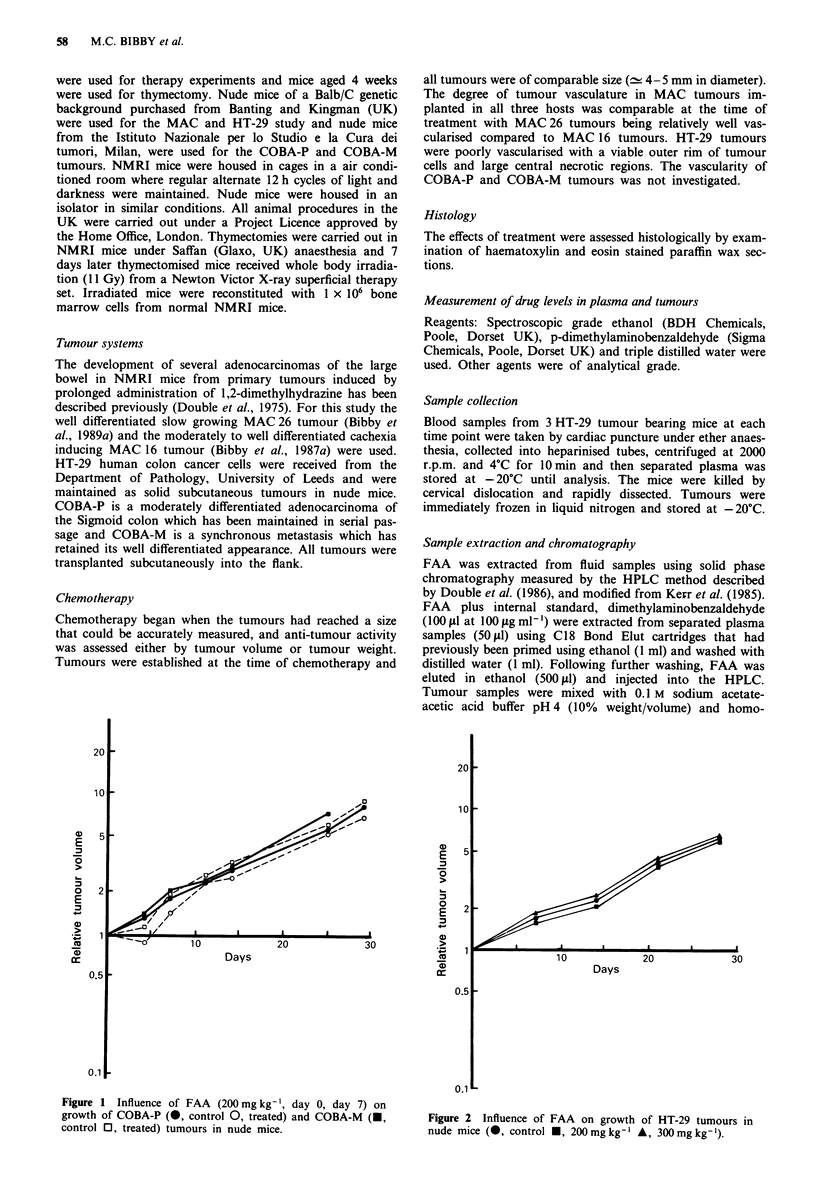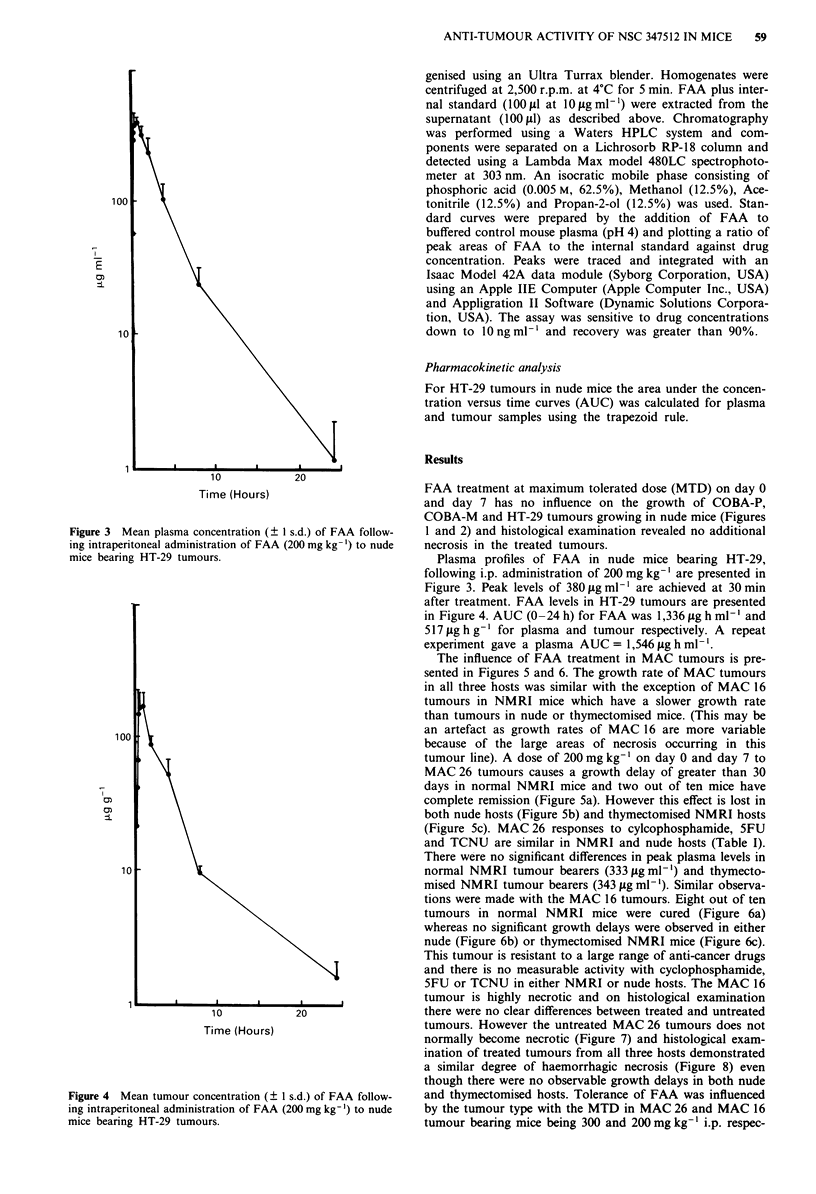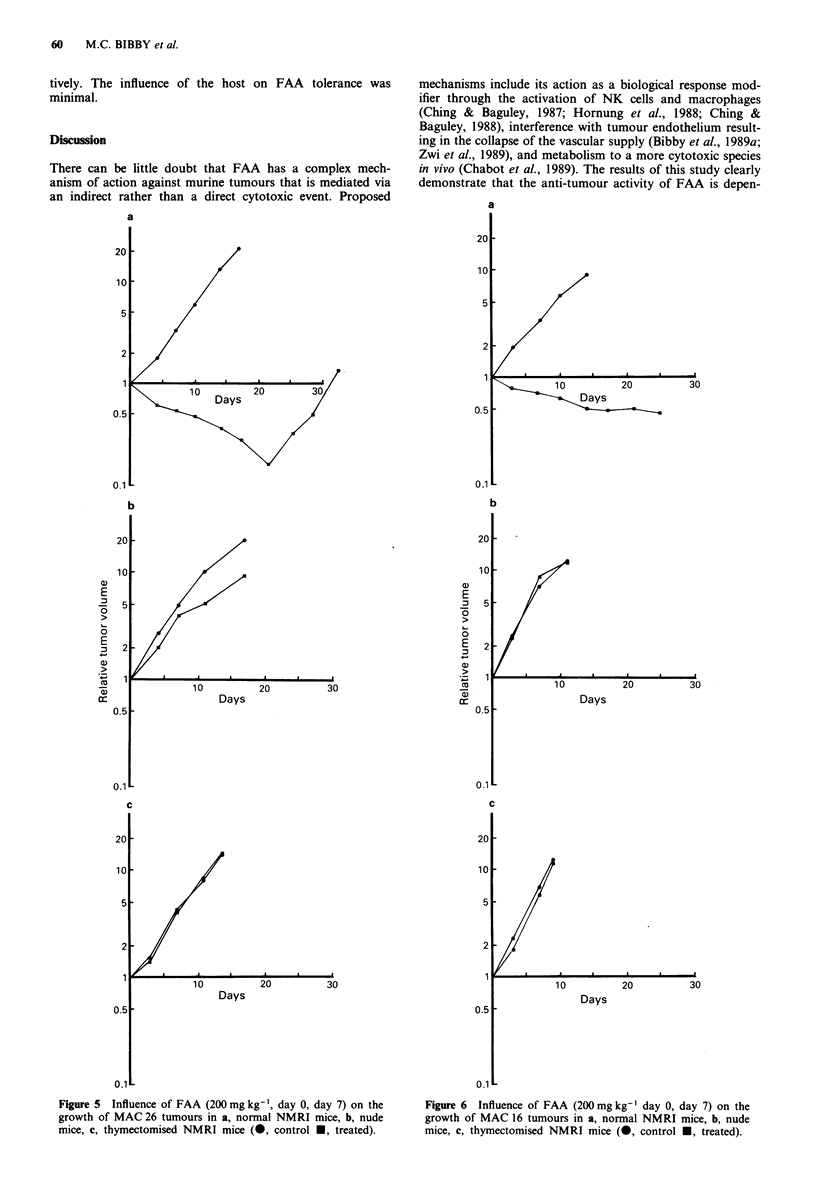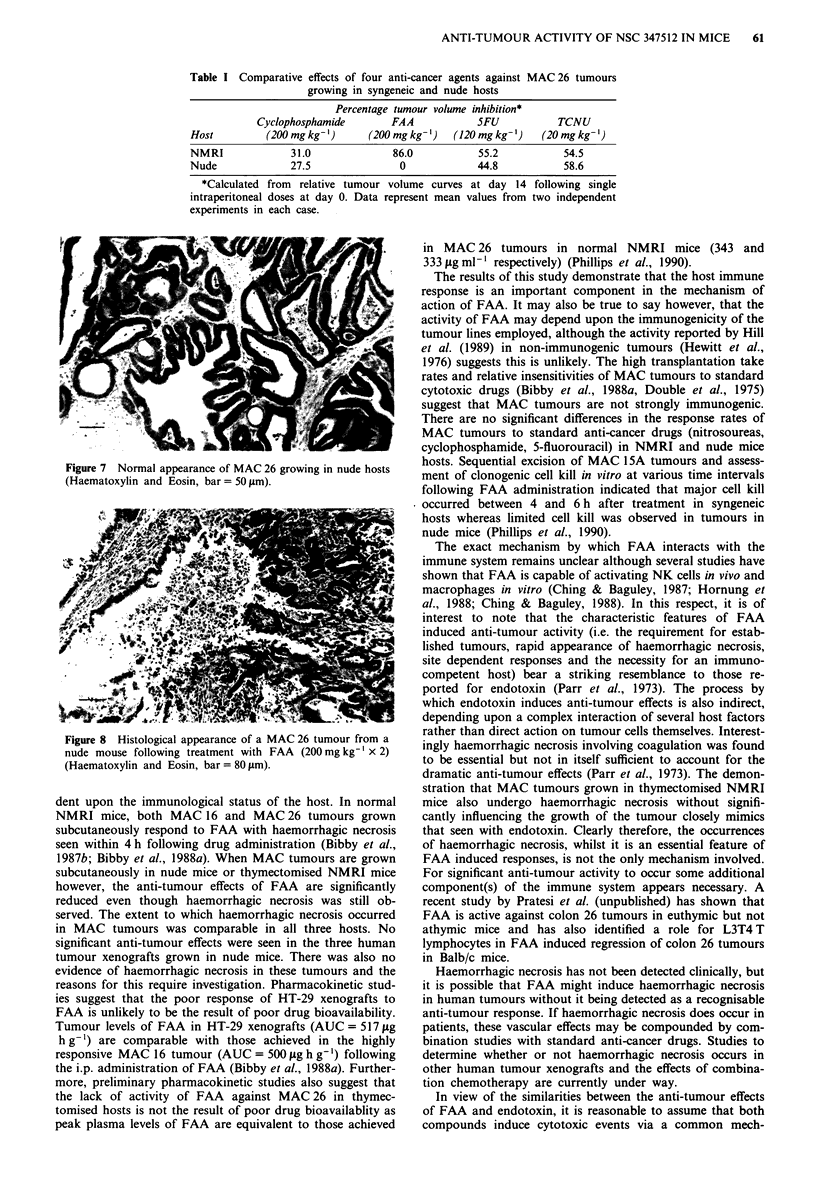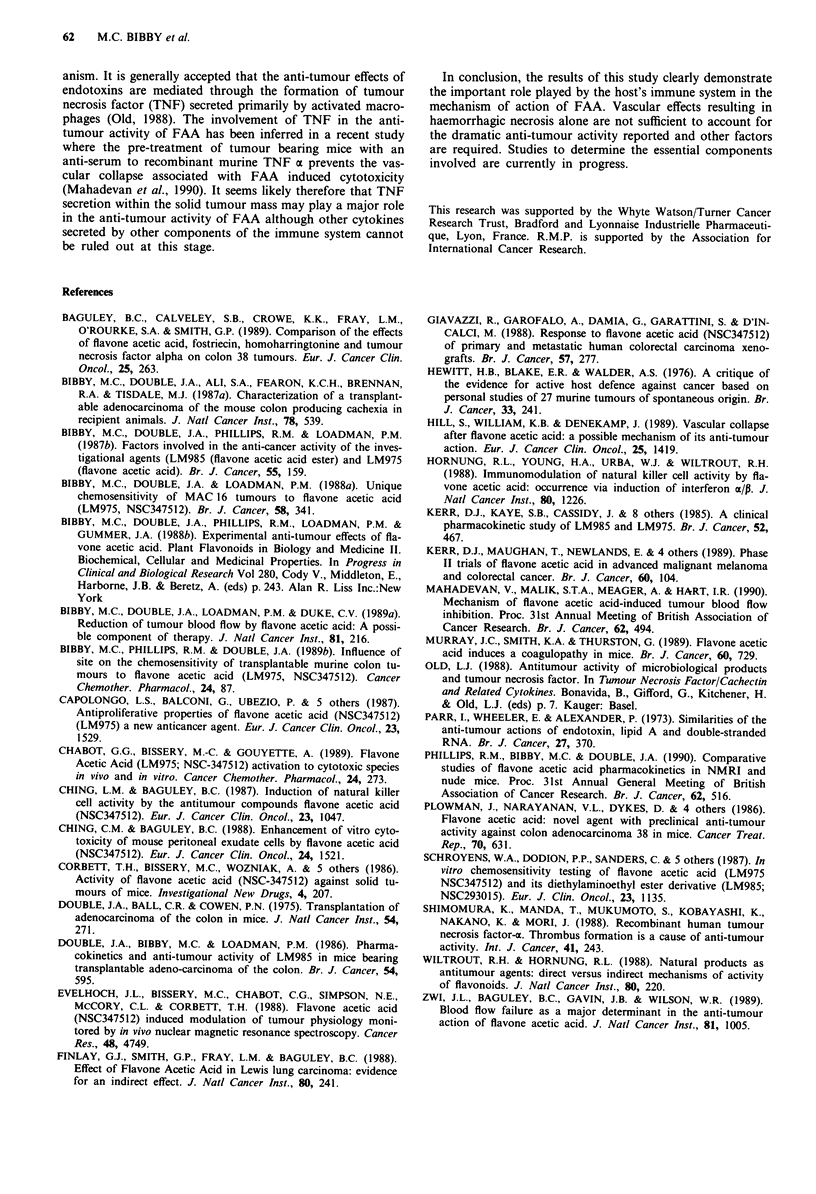# Anti-tumour activity of flavone acetic acid (NSC 347512) in mice--influence of immune status.

**DOI:** 10.1038/bjc.1991.12

**Published:** 1991-01

**Authors:** M. C. Bibby, R. M. Phillips, J. A. Double, G. Pratesi

**Affiliations:** Clinical Oncology Unit, University of Bradford, West Yorkshire, UK.

## Abstract

**Images:**


Br. J. Cancer (1991), 63, 57-62 © Macmillan Press Ltd., 1991

### Anti-tumour activity of flavone acetic acid (NSC 347512) in mice – influence of immune status

M.C. Bibby[1], R.M. Phillips[1], J.A. Double[1,*] & G. Pratesi[2,*]

[1]*Clinical Oncology Unit, University of Bradford, Bradford BD7 1DP, West Yorkshire, UK; and* [2]*Experimental Oncology B, Istituto Nazionale per lo Studio e la Cura dei tumori, Via Venezian 1, 20133, Milan, Italy.*

**Summary** Flavone acetic acid (FAA) is a synthetic flavonoid with dramatic pre-clinical anti-tumour activity involving a vascular component in its mechanism but no clinical effects have been seen to date. As FAA also has immunomodulatory activity, immunological factors might explain differences in activity between mouse and man. This study examines the influence of host immune status on the anti-tumour activity of FAA. Two human colon tumour xenografts (COBA, HT-29) fail to respond to FAA in nude mice. The lack of activity of FAA against HT-29 xenografts cannot be explained on the basis of limited drug bioavailability as achievable plasma, and tumour levels of FAA are similar to those seen in sensitive murine colon tumours. The immune status of the host also influences the activity of FAA against two transplantable tumours of the mouse colon. Both these tumours are highly responsive to FAA in their normal NMRI hosts, but neither tumours exhibited significant growth delay in thymectomised NMRI or nude hosts. Histological examination of treated tumours revealed significant areas of haemorrhagic necrosis in all three hosts. These data suggest a clear immunological component in the mechanism of action of FAA which is separate from the previously described haemorrhagic necrosis.

Flavone acetic acid (FAA) is a synthetic flavonoid with interesting pre-clinical activity against a broad spectrum of murine transplantable solid tumours that are refractory to conventional cytotoxic agents (Corbett *et al.*, 1986; Plowman *et al.*, 1986; Bibby *et al.*, 1987*b*; Bibby *et al.*, 1988*a*). Despite these promising pre-clinical observations, FAA has no demonstrable clinical activity (Kerr *et al.*, 1989). Previous *in vitro* studies with a variety of tumour cell lines have indicated that high drug concentrations, or long exposure times, are necessary to achieve direct drug cytotoxicity with FAA (Bibby *et al.*, 1987; Capolongo *et al.*, 1987; Schroyens *et al.*, 1987). These drug profiles are not achieved in mice *in vivo* suggesting that the anti-tumour effects against subcutaneous mouse tumours are not the result of a direct cytotoxic mechanism.

Numerous possible mechanisms have been proposed in order to explain this indirect activity and most of these suggest the involvement of a critical host component. A number of studies has demonstrated that FAA has immunomodulatory activity (Ching & Baguley, 1987; Hornung *et al.*, 1988; Wiltrout & Hornung, 1988; Ching & Baguley, 1988). Earlier studies in this laboratory have demonstrated that the establishment of a tumour vasculature may be necessary for the achievement of responses (Bibby *et al.*, 1988*b*). More recent studies have shown that anti-tumour activity in subcutaneous tumours is accompanied by vascular shut-down and a reduction in tumour blood flow (Bibby *et al.*, 1989*a*; Evelhoch *et al.*, 1988; Zwi *et al.*, 1989). Further investigations have shown that following FAA treatment in mice, clotting times were significantly reduced suggesting that possible intravascular coagulation occurs resulting in the vascular occlusion of tumours (Murray *et al.*, 1989). Although inhibition of tumour blood flow by FAA is an obvious component of its anti-tumour activity in solid subcutaneous tumours it is still necessary to establish whether this is the key mechanism of action. It is certainly an attractive possibility because systemic tumours in mice which do not develop a capillary blood vasculature do not respond to FAA (Bibby *et al.*, 1989*b*).

The anti-tumour activity of FAA in subcutaneous tumours is accompanied by histological changes similar to those produced by tumour necrosis factor (TNF) (Baguley *et al.*, 1989). TNF has been shown to produce thrombus formation which may be involved in its anti-tumour activity (Shimomura *et al.*, 1988). The histological appearance of subcutaneous tumours treated with FAA is also very similar to that reported following endotoxin treatment (Parr *et al.*, 1973). The tumour regression brought about by endotoxin was thought to be due to vascular damage to the tumour which permitted access for the immune defence mechanisms of the host to the tumour and also to activation of macrophages present within the tumour. Immunosuppression interfered with the anti-tumour action of endotoxin in spite of the fact that haemorrhagic necrosis still occurred. Response of human tumour xenografts to FAA appears to be modest (Giavazzi *et al.*, 1988; Finlay *et al.*, 1988; H. Fiebig personal communication) and there are certainly no published data demonstrating spectacular responses similar to those achieved in subcutaneous murine tumours.

The aims of this present study are to examine the influence of FAA on the growth of human colon tumour xenografts (HT29, COBA) in immune suppressed mice and also to compare the pharmacokinetics of FAA in nude mice with previously published mouse data (Bibby *et al.*, 1987*b*; Bibby *et al.*, 1988*a*; Bibby *et al.*, 1989*a*). To further study the influence of immune status the investigation also compares the effects of FAA on mouse adenocarcinomas of the colon (MAC tumours) in their syngeneic hosts with the effects achieved in nude and thymectomised hosts.

### Materials and methods

### *Drugs*

FAA was supplied as pure compound by the NCI (Bethesda) for the Milan study and dissolved in 5% sodium bicarbonate. For the Bradford study, clinically formulated FAA was a gift from Lyonnaise Industrielle Pharmaceutique (Lyon, France). Formulated FAA was dissolved in physiological saline. Cyclophosphamide, 5 Fluorouracil (5FU) and tauromustine (TCNU) were gifts from the Boehringer Corporation, London: Roche, Welwyn Garden City, UK and Pharmacia LEO Therapeutics AB, Helsingborg, Sweden, respectively. They were dissolved in physiological saline. All preparations were at an appropriate concentration for the desired dose to be administered in 0.1 ml per 10 g body weight. All injections were intraperitoneal (i.p.).

### *Animals*

Pure strain NMRI mice were used from the Bradford Clinical Oncology Unit inbred colony. Mice aged 6–8 weeks

Correspondence: M.C. Bibby.
*Screening and Pharmacology Group, EORTC.
Received 6 July 1990; and in revised form 12 September 1990.

were used for therapy experiments and mice aged 4 weeks were used for thymectomy. Nude mice of a Balb/C genetic background purchased from Banting and Kingman (UK) were used for the MAC and HT-29 study and nude mice from the Istituto Nazionale per lo Studio e la Cura dei tumori, Milan, were used for the COBA-P and COBA-M tumours. NMRI mice were housed in cages in a air conditioned room where regular alternate 12 h cycles of light and darkness were maintained. Nude mice were housed in an isolator in similar conditions. All animal procedures in the UK were carried out under a Project Licence approved by the Home Office, London. Thymectomies were carried out in NMRI mice under Saffan (Glaxo, UK) anaesthesia and 7 days later thymectomised mice received whole body irradiation (11 Gy) from a Newton Victor X-ray superficial therapy set. Irradiated mice were reconstituted with $1 \times 10^6$ bone marrow cells from normal NMRI mice.

*Tumour systems*

The development of several adenocarcinomas of the large bowel in NMRI mice from primary tumours induced by prolonged administration of 1,2-dimethylhydrazine has been described previously (Double *et al.*, 1975). For this study the well differentiated slow growing MAC 26 tumour (Bibby *et al.*, 1989*a*) and the moderately to well differentiated cachexia inducing MAC 16 tumour (Bibby *et al.*, 1987*a*) were used. HT-29 human colon cancer cells were received from the Department of Pathology, University of Leeds and were maintained as solid subcutaneous tumours in nude mice. COBA-P is a moderately differentiated adenocarcinoma of the Sigmoid colon which has been maintained in serial passage and COBA-M is a synchronous metastasis which has retained its well differentiated appearance. All tumours were transplanted subcutaneously into the flank.

*Chemotherapy*

Chemotherapy began when the tumours had reached a size that could be accurately measured, and anti-tumour activity was assessed either by tumour volume or tumour weight. Tumours were established at the time of chemotherapy and all tumours were of comparable size (≃ 4–5 mm in diameter). The degree of tumour vasculature in MAC tumours implanted in all three hosts was comparable at the time of treatment with MAC 26 tumours being relatively well vascularised compared to MAC 16 tumours. HT-29 tumours were poorly vascularised with a viable outer rim of tumour cells and large central necrotic regions. The vascularity of COBA-P and COBA-M tumours was not investigated.

*Histology*

The effects of treatment were assessed histologically by examination of haematoxylin and eosin stained paraffin wax sections.

*Measurement of drug levels in plasma and tumours*

Reagents: Spectroscopic grade ethanol (BDH Chemicals, Poole, Dorset UK), p-dimethylaminobenzaldehyde (Sigma Chemicals, Poole, Dorset UK) and triple distilled water were used. Other agents were of analytical grade.

*Sample collection*

Blood samples from 3 HT-29 tumour bearing mice at each time point were taken by cardiac puncture under ether anaesthesia, collected into heparinised tubes, centrifuged at 2000 r.p.m. and 4°C for 10 min and then separated plasma was stored at −20°C until analysis. The mice were killed by cervical dislocation and rapidly dissected. Tumours were immediately frozen in liquid nitrogen and stored at −20°C.

*Sample extraction and chromatography*

FAA was extracted from fluid samples using solid phase chromatography measured by the HPLC method described by Double *et al.* (1986), and modified from Kerr *et al.* (1985). FAA plus internal standard, dimethylaminobenzaldehyde (100 µl at 100 µg ml$^{-1}$) were extracted from separated plasma samples (50 µl) using C18 Bond Elut cartridges that had previously been primed using ethanol (1 ml) and washed with distilled water (1 ml). Following further washing, FAA was eluted in ethanol (500 µl) and injected into the HPLC. Tumour samples were mixed with 0.1 M sodium acetate-acetic acid buffer pH 4 (10% weight/volume) and homo-

**Figure 1** Influence of FAA (200 mg kg$^{-1}$, day 0, day 7) on growth of COBA-P (●, control ○, treated) and COBA-M (■, control □, treated) tumours in nude mice.

**Figure 2** Influence of FAA on growth of HT-29 tumours in nude mice (●, control ■, 200 mg kg$^{-1}$ ▲, 300 mg kg$^{-1}$).

**Figure 3** Mean plasma concentration (± 1 s.d.) of FAA following intraperitoneal administration of FAA (200 mg kg$^{-1}$) to nude mice bearing HT-29 tumours.

**Figure 4** Mean tumour concentration (± 1 s.d.) of FAA following intraperitoneal administration of FAA (200 mg kg$^{-1}$) to nude mice bearing HT-29 tumours.

genised using an Ultra Turrax blender. Homogenates were centrifuged at 2,500 r.p.m. at 4°C for 5 min. FAA plus internal standard (100 μl at 10 μg ml$^{-1}$) were extracted from the supernatant (100 μl) as described above. Chromatography was performed using a Waters HPLC system and components were separated on a Lichrosorb RP-18 column and detected using a Lambda Max model 480LC spectrophotometer at 303 nm. An isocratic mobile phase consisting of phosphoric acid (0.005 M, 62.5%), Methanol (12.5%), Acetonitrile (12.5%) and Propan-2-ol (12.5%) was used. Standard curves were prepared by the addition of FAA to buffered control mouse plasma (pH 4) and plotting a ratio of peak areas of FAA to the internal standard against drug concentration. Peaks were traced and integrated with an Isaac Model 42A data module (Syborg Corporation, USA) using an Apple IIE Computer (Apple Computer Inc., USA) and Appligration II Software (Dynamic Solutions Corporation, USA). The assay was sensitive to drug concentrations down to 10 ng ml$^{-1}$ and recovery was greater than 90%.

*Pharmacokinetic analysis*

For HT-29 tumours in nude mice the area under the concentration versus time curves (AUC) was calculated for plasma and tumour samples using the trapezoid rule.

### Results

FAA treatment at maximum tolerated dose (MTD) on day 0 and day 7 has no influence on the growth of COBA-P, COBA-M and HT-29 tumours growing in nude mice (Figures 1 and 2) and histological examination revealed no additional necrosis in the treated tumours.

Plasma profiles of FAA in nude mice bearing HT-29, following i.p. administration of 200 mg kg$^{-1}$ are presented in Figure 3. Peak levels of 380 μg ml$^{-1}$ are achieved at 30 min after treatment. FAA levels in HT-29 tumours are presented in Figure 4. AUC (0–24 h) for FAA was 1,336 μg h ml$^{-1}$ and 517 μg h g$^{-1}$ for plasma and tumour respectively. A repeat experiment gave a plasma AUC = 1,546 μg h ml$^{-1}$.

The influence of FAA treatment in MAC tumours is presented in Figures 5 and 6. The growth rate of MAC tumours in all three hosts was similar with the exception of MAC 16 tumours in NMRI mice which have a slower growth rate than tumours in nude or thymectomised mice. (This may be an artefact as growth rates of MAC 16 are more variable because of the large areas of necrosis occurring in this tumour line). A dose of 200 mg kg$^{-1}$ on day 0 and day 7 to MAC 26 tumours causes a growth delay of greater than 30 days in normal NMRI mice and two out of ten mice have complete remission (Figure 5a). However this effect is lost in both nude hosts (Figure 5b) and thymectomised NMRI hosts (Figure 5c). MAC 26 responses to cylcophosphamide, 5FU and TCNU are similar in NMRI and nude hosts (Table I). There were no significant differences in peak plasma levels in normal NMRI tumour bearers (333 μg ml$^{-1}$) and thymectomised NMRI tumour bearers (343 μg ml$^{-1}$). Similar observations were made with the MAC 16 tumours. Eight out of ten tumours in normal NMRI mice were cured (Figure 6a) whereas no significant growth delays were observed in either nude (Figure 6b) or thymectomised NMRI mice (Figure 6c). This tumour is resistant to a large range of anti-cancer drugs and there is no measurable activity with cyclophosphamide, 5FU or TCNU in either NMRI or nude hosts. The MAC 16 tumour is highly necrotic and on histological examination there were no clear differences between treated and untreated tumours. However the untreated MAC 26 tumours does not normally become necrotic (Figure 7) and histological examination of treated tumours from all three hosts demonstrated a similar degree of haemorrhagic necrosis (Figure 8) even though there were no observable growth delays in both nude and thymectomised hosts. Tolerance of FAA was influenced by the tumour type with the MTD in MAC 26 and MAC 16 tumour bearing mice being 300 and 200 mg kg$^{-1}$ i.p. respectively. The influence of the host on FAA tolerance was minimal.

### Discussion

There can be little doubt that FAA has a complex mechanism of action against murine tumours that is mediated via an indirect rather than a direct cytotoxic event. Proposed mechanisms include its action as a biological response modifier through the activation of NK cells and macrophages (Ching & Baguley, 1987; Hornung *et al.*, 1988; Ching & Baguley, 1988), interference with tumour endothelium resulting in the collapse of the vascular supply (Bibby *et al.*, 1989*a*; Zwi *et al.*, 1989), and metabolism to a more cytotoxic species *in vivo* (Chabot *et al.*, 1989). The results of this study clearly demonstrate that the anti-tumour activity of FAA is depen-

**Figure 5** Influence of FAA (200 mg kg$^{-1}$, day 0, day 7) on the growth of MAC 26 tumours in **a**, normal NMRI mice, **b**, nude mice, **c**, thymectomised NMRI mice (●, control ■, treated).

**Figure 6** Influence of FAA (200 mg kg$^{-1}$ day 0, day 7) on the growth of MAC 16 tumours in **a**, normal NMRI mice, **b**, nude mice, **c**, thymectomised NMRI mice (●, control ■, treated).

**Table I** Comparative effects of four anti-cancer agents against MAC 26 tumours growing in syngeneic and nude hosts

| Host | *Percentage tumour volume inhibition** Cyclophosphamide (200 mg kg$^{-1}$) | FAA (200 mg kg$^{-1}$) | 5FU (120 mg kg$^{-1}$) | TCNU (20 mg kg$^{-1}$) |
|---|---|---|---|---|
| NMRI | 31.0 | 86.0 | 55.2 | 54.5 |
| Nude | 27.5 | 0 | 44.8 | 58.6 |

*Calculated from relative tumour volume curves at day 14 following single intraperitoneal doses at day 0. Data represent mean values from two independent experiments in each case.

**Figure 7** Normal appearance of MAC 26 growing in nude hosts (Haematoxylin and Eosin, bar = 50 μm).

**Figure 8** Histological appearance of a MAC 26 tumour from a nude mouse following treatment with FAA (200 mg kg$^{-1}$ × 2) (Haematoxylin and Eosin, bar = 80 μm).

dent upon the immunological status of the host. In normal NMRI mice, both MAC 16 and MAC 26 tumours grown subcutaneously respond to FAA with haemorrhagic necrosis seen within 4 h following drug administration (Bibby *et al.*, 1987*b*; Bibby *et al.*, 1988*a*). When MAC tumours are grown subcutaneously in nude mice or thymectomised NMRI mice however, the anti-tumour effects of FAA are significantly reduced even though haemorrhagic necrosis was still observed. The extent to which haemorrhagic necrosis occurred in MAC tumours was comparable in all three hosts. No significant anti-tumour effects were seen in the three human tumour xenografts grown in nude mice. There was also no evidence of haemorrhagic necrosis in these tumours and the reasons for this require investigation. Pharmacokinetic studies suggest that the poor response of HT-29 xenografts to FAA is unlikely to be the result of poor drug bioavailability. Tumour levels of FAA in HT-29 xenografts (AUC = 517 μg h g$^{-1}$) are comparable with those achieved in the highly responsive MAC 16 tumour (AUC = 500 μg h g$^{-1}$) following the i.p. administration of FAA (Bibby *et al.*, 1988*a*). Furthermore, preliminary pharmacokinetic studies also suggest that the lack of activity of FAA against MAC 26 in thymectomised hosts is not the result of poor drug bioavailablity as peak plasma levels of FAA are equivalent to those achieved in MAC 26 tumours in normal NMRI mice (343 and 333 μg ml$^{-1}$ respectively) (Phillips *et al.*, 1990).

The results of this study demonstrate that the host immune response is an important component in the mechanism of action of FAA. It may also be true to say however, that the activity of FAA may depend upon the immunogenicity of the tumour lines employed, although the activity reported by Hill *et al.* (1989) in non-immunogenic tumours (Hewitt *et al.*, 1976) suggests this is unlikely. The high transplantation take rates and relative insensitivities of MAC tumours to standard cytotoxic drugs (Bibby *et al.*, 1988*a*, Double *et al.*, 1975) suggest that MAC tumours are not strongly immunogenic. There are no significant differences in the response rates of MAC tumours to standard anti-cancer drugs (nitrosoureas, cyclophosphamide, 5-fluorouracil) in NMRI and nude mice hosts. Sequential excision of MAC 15A tumours and assessment of clonogenic cell kill *in vitro* at various time intervals following FAA administration indicated that major cell kill occurred between 4 and 6 h after treatment in syngeneic hosts whereas limited cell kill was observed in tumours in nude mice (Phillips *et al.*, 1990).

The exact mechanism by which FAA interacts with the immune system remains unclear although several studies have shown that FAA is capable of activating NK cells *in vivo* and macrophages *in vitro* (Ching & Baguley, 1987; Hornung *et al.*, 1988; Ching & Baguley, 1988). In this respect, it is of interest to note that the characteristic features of FAA induced anti-tumour activity (i.e. the requirement for established tumours, rapid appearance of haemorrhagic necrosis, site dependent responses and the necessity for an immunocompetent host) bear a striking resemblance to those reported for endotoxin (Parr *et al.*, 1973). The process by which endotoxin induces anti-tumour effects is also indirect, depending upon a complex interaction of several host factors rather than direct action on tumour cells themselves. Interestingly haemorrhagic necrosis involving coagulation was found to be essential but not in itself sufficient to account for the dramatic anti-tumour effects (Parr *et al.*, 1973). The demonstration that MAC tumours grown in thymectomised NMRI mice also undergo haemorrhagic necrosis without significantly influencing the growth of the tumour closely mimics that seen with endotoxin. Clearly therefore, the occurrences of haemorrhagic necrosis, whilst it is an essential feature of FAA induced responses, is not the only mechanism involved. For significant anti-tumour activity to occur some additional component(s) of the immune system appears necessary. A recent study by Pratesi *et al.* (unpublished) has shown that FAA is active against colon 26 tumours in euthymic but not athymic mice and has also identified a role for L3T4 T lymphocytes in FAA induced regression of colon 26 tumours in Balb/c mice.

Haemorrhagic necrosis has not been detected clinically, but it is possible that FAA might induce haemorrhagic necrosis in human tumours without it being detected as a recognisable anti-tumour response. If haemorrhagic necrosis does occur in patients, these vascular effects may be compounded by combination studies with standard anti-cancer drugs. Studies to determine whether or not haemorrhagic necrosis occurs in other human tumour xenografts and the effects of combination chemotherapy are currently under way.

In view of the similarities between the anti-tumour effects of FAA and endotoxin, it is reasonable to assume that both compounds induce cytotoxic events via a common mechanism. It is generally accepted that the anti-tumour effects of endotoxins are mediated through the formation of tumour necrosis factor (TNF) secreted primarily by activated macrophages (Old, 1988). The involvement of TNF in the anti-tumour activity of FAA has been inferred in a recent study where the pre-treatment of tumour bearing mice with an anti-serum to recombinant murine TNF α prevents the vascular collapse associated with FAA induced cytotoxicity (Mahadevan *et al.*, 1990). It seems likely therefore that TNF secretion within the solid tumour mass may play a major role in the anti-tumour activity of FAA although other cytokines secreted by other components of the immune system cannot be ruled out at this stage.

In conclusion, the results of this study clearly demonstrate the important role played by the host's immune system in the mechanism of action of FAA. Vascular effects resulting in haemorrhagic necrosis alone are not sufficient to account for the dramatic anti-tumour activity reported and other factors are required. Studies to determine the essential components involved are currently in progress.

This research was supported by the Whyte Watson/Turner Cancer Research Trust, Bradford and Lyonnaise Industrielle Pharmaceutique, Lyon, France. R.M.P. is supported by the Association for International Cancer Research.

### References

BAGULEY, B.C., CALVELEY, S.B., CROWE, K.K., FRAY, L.M., O'ROURKE, S.A. & SMITH, G.P. (1989). Comparison of the effects of flavone acetic acid, fostriecin, homoharringtonine and tumour necrosis factor alpha on colon 38 tumours. *Eur. J. Cancer Clin. Oncol.*, **25**, 263.

BIBBY, M.C., DOUBLE, J.A., ALI, S.A., FEARON, K.C.H., BRENNAN, R.A. & TISDALE, M.J. (1987*a*). Characterization of a transplantable adenocarcinoma of the mouse colon producing cachexia in recipient animals. *J. Natl Cancer Inst.*, **78**, 539.

BIBBY, M.C., DOUBLE, J.A., PHILLIPS, R.M. & LOADMAN, P.M. (1987*b*). Factors involved in the anti-cancer activity of the investigational agents (LM985 (flavone acetic acid ester) and LM975 (flavone acetic acid). *Br. J. Cancer*, **55**, 159.

BIBBY, M.C., DOUBLE, J.A. & LOADMAN, P.M. (1988*a*). Unique chemosensitivity of MAC 16 tumours to flavone acetic acid (LM975, NSC347512). *Br. J. Cancer*, **58**, 341.

BIBBY, M.C., DOUBLE, J.A., PHILLIPS, R.M., LOADMAN, P.M. & GUMMER, J.A. (1988*b*). Experimental anti-tumour effects of flavone acetic acid. Plant Flavonoids in Biology and Medicine II. Biochemical, Cellular and Medicinal Properties. In *Progress in Clinical and Biological Research* Vol 280, Cody V., Middleton, E., Harborne, J.B. & Beretz, A. (eds) p. 243. Alan R. Liss Inc.:New York

BIBBY, M.C., DOUBLE, J.A., LOADMAN, P.M. & DUKE, C.V. (1989*a*). Reduction of tumour blood flow by flavone acetic acid: A possible component of therapy. *J. Natl Cancer Inst.*, **81**, 216.

BIBBY, M.C., PHILLIPS, R.M. & DOUBLE, J.A. (1989*b*). Influence of site on the chemosensitivity of transplantable murine colon tumours to flavone acetic acid (LM975, NSC347512). *Cancer Chemother. Pharmacol.*, **24**, 87.

CAPOLONGO, L.S., BALCONI, G., UBEZIO, P. & 5 others (1987). Antiproliferative properties of flavone acetic acid (NSC347512) (LM975) a new anticancer agent. *Eur. J. Cancer Clin. Oncol.*, **23**, 1529.

CHABOT, G.G., BISSERY, M.-C. & GOUYETTE, A. (1989). Flavone Acetic Acid (LM975; NSC-347512) activation to cytotoxic species *in vivo* and *in vitro*. *Cancer Chemother. Pharmacol.*, **24**, 273.

CHING, L.M. & BAGULEY, B.C. (1987). Induction of natural killer cell activity by the antitumour compounds flavone acetic acid (NSC347512). *Eur. J. Cancer Clin. Oncol.*, **23**, 1047.

CHING, C.M. & BAGULEY, B.C. (1988). Enhancement of vitro cytotoxicity of mouse peritoneal exudate cells by flavone acetic acid (NSC347512). *Eur. J. Cancer Clin. Oncol.*, **24**, 1521.

CORBETT, T.H., BISSERY, M.C., WOZNIAK, A. & 5 others (1986). Activity of flavone acetic acid (NSC-347512) against solid tumours of mice. *Investigational New Drugs*, **4**, 207.

DOUBLE, J.A., BALL, C.R. & COWEN, P.N. (1975). Transplantation of adenocarcinoma of the colon in mice. *J. Natl Cancer Inst.*, **54**, 271.

DOUBLE, J.A., BIBBY, M.C. & LOADMAN, P.M. (1986). Pharmacokinetics and anti-tumour activity of LM985 in mice bearing transplantable adeno-carcinoma of the colon. *Br. J. Cancer*, **54**, 595.

EVELHOCH, J.L., BISSERY, M.C., CHABOT, C.G., SIMPSON, N.E., MCCORY, C.L. & CORBETT, T.H. (1988). Flavone acetic acid (NSC347512) induced modulation of tumour physiology monitored by *in vivo* nuclear magnetic resonance spectroscopy. *Cancer Res.*, **48**, 4749.

FINLAY, G.J., SMITH, G.P., FRAY, L.M. & BAGULEY, B.C. (1988). Effect of Flavone Acetic Acid in Lewis lung carcinoma: evidence for an indirect effect. *J. Natl Cancer Inst.*, **80**, 241.

GIAVAZZI, R., GAROFALO, A., DAMIA, G., GARATTINI, S. & D'INCALCI, M. (1988). Response to flavone acetic acid (NSC347512) of primary and metastatic human colorectal carcinoma xenografts. *Br. J. Cancer*, **57**, 277.

HEWITT, H.B., BLAKE, E.R. & WALDER, A.S. (1976). A critique of the evidence for active host defence against cancer based on personal studies of 27 murine tumours of spontaneous origin. *Br. J. Cancer*, **33**, 241.

HILL, S., WILLIAM, K.B. & DENEKAMP, J. (1989). Vascular collapse after flavone acetic acid: a possible mechanism of its anti-tumour action. *Eur. J. Cancer Clin. Oncol.*, **25**, 1419.

HORNUNG, R.L., YOUNG, H.A., URBA, W.J. & WILTROUT, R.H. (1988). Immunomodulation of natural killer cell activity by flavone acetic acid: occurrence via induction of interferon α/β. *J. Natl Cancer Inst.*, **80**, 1226.

KERR, D.J., KAYE, S.B., CASSIDY, J. & 8 others (1985). A clinical pharmacokinetic study of LM985 and LM975. *Br. J. Cancer*, **52**, 467.

KERR, D.J., MAUGHAN, T., NEWLANDS, E. & 4 others (1989). Phase II trials of flavone acetic acid in advanced malignant melanoma and colorectal cancer. *Br. J. Cancer*, **60**, 104.

MAHADEVAN, V., MALIK, S.T.A., MEAGER, A. & HART, I.R. (1990). Mechanism of flavone acetic acid-induced tumour blood flow inhibition. Proc. 31st Annual Meeting of British Association of Cancer Research. *Br. J. Cancer*, **62**, 494.

MURRAY, J.C., SMITH, K.A. & THURSTON, G. (1989). Flavone acetic acid induces a coagulopathy in mice. *Br. J. Cancer*, **60**, 729.

OLD, L.J. (1988). Antitumour activity of microbiological products and tumour necrosis factor. In *Tumour Necrosis Factor/Cachectin and Related Cytokines*. Bonavida, B., Gifford, G., Kitchener, H. & Old, L.J. (eds) p. 7. Kauger: Basel.

PARR, I., WHEELER, E. & ALEXANDER, P. (1973). Similarities of the anti-tumour actions of endotoxin, lipid A and double-stranded RNA. *Br. J. Cancer*, **27**, 370.

PHILLIPS, R.M., BIBBY, M.C. & DOUBLE, J.A. (1990). Comparative studies of flavone acetic acid pharmacokinetics in NMRI and nude mice. Proc. 31st Annual General Meeting of British Association of Cancer Research. *Br. J. Cancer*, **62**, 516.

PLOWMAN, J., NARAYANAN, V.L., DYKES, D. & 4 others (1986). Flavone acetic acid: novel agent with preclinical anti-tumour activity against colon adenocarcinoma 38 in mice. *Cancer Treat. Rep.*, **70**, 631.

SCHROYENS, W.A., DODION, P.P., SANDERS, C. & 5 others (1987). *In vitro* chemosensitivity testing of flavone acetic acid (LM975 NSC347512) and its diethylaminoethyl ester derivative (LM985; NSC293015). *Eur. J. Clin. Oncol.*, **23**, 1135.

SHIMOMURA, K., MANDA, T., MUKUMOTO, S., KOBAYASHI, K., NAKANO, K. & MORI, J. (1988). Recombinant human tumour necrosis factor-α. Thrombus formation is a cause of anti-tumour activity. *Int. J. Cancer*, **41**, 243.

WILTROUT, R.H. & HORNUNG, R.L. (1988). Natural products as antitumour agents: direct versus indirect mechanisms of activity of flavonoids. *J. Natl Cancer Inst.*, **80**, 220.

ZWI, J.L., BAGULEY, B.C., GAVIN, J.B. & WILSON, W.R. (1989). Blood flow failure as a major determinant in the anti-tumour action of flavone acetic acid. *J. Natl Cancer Inst.*, **81**, 1005.